# Pathogen-induced *Caenorhabditis elegans* developmental plasticity has a hormetic effect on the resistance to biotic and abiotic stresses

**DOI:** 10.1186/1471-2148-12-187

**Published:** 2012-09-21

**Authors:** Magali Leroy, Thomas Mosser, Xavier Manière, Diana Fernández Alvarez, Ivan Matic

**Affiliations:** 1 Laboratory of Evolutive, Medical and Molecular Genetics, Inserm U1001, Université Paris Descartes, Sorbonne Paris Cité, Faculté De Médecine Paris Descartes, 156 rue de Vaugirard, Paris, 75730 cedex 15, France

**Keywords:** Caenorhabditis elegans, Development, Lifespan, Stress resistance, Hormesis, Pathogens

## Abstract

**Background:**

Phenotypic plasticity, i.e. the capacity to change the phenotype in response to changes in the environment without alteration of the genotype, is important for coping with unstable environments. In spite of the ample evidence that microorganisms are a major environmental component playing a significant role in eukaryotic organisms health and disease, there is not much information about the effect of microorganism-induced developmental phenotypic plasticity on adult animals’ stress resistance and longevity.

**Results:**

We examined the consequences of development of *Caenorhabditis elegans* larvae fed with different bacterial strains on stress resistance and lifespan of adult nematodes. Bacterial strains used in this study were either pathogenic or innocuous to nematodes. Exposure to the pathogen during development did not affect larval survival. However, the development of nematodes on the pathogenic bacterial strains increased lifespan of adult nematodes exposed to the same or a different pathogen. A longer nematode lifespan, developed on pathogens and exposed to pathogens as adults, did not result from an enhanced capacity to kill bacteria, but is likely due to an increased tolerance to the damage inflicted by the pathogenic bacteria. We observed that adult nematodes developed on a pathogen induce higher level of expression of the *hsp-16.2* gene and have higher resistance to heat shock than nematodes developed on an innocuous strain. Therefore, the increased resistance to pathogens could be, at least partially, due to the early induction of the heat shock response in nematodes developed on pathogens. The lifespan increase is controlled by the DBL-1 transforming growth factor beta-like, DAF-2/DAF-16 insulin-like, and p38 MAP kinase pathways. Therefore, the observed modulation of adult nematode lifespans by developmental exposure to a pathogen is likely a genetically controlled response.

**Conclusions:**

Our study shows that development on pathogens has a hormetic effect on adult nematodes, as it results in increased resistance to different pathogens and to heat shock. Such developmental plasticity of *C. elegans* nematodes, which are self-fertilizing homozygous animals producing offspring with negligible genetic variation, could increase the probability of survival in changing environments.

## Background

Multicellular organisms have evolve powerful controlling mechanisms to cope with diverse perturbations in order to assure phenotypically quasi-invariant developmental outcome [1]. However, development can be influenced by environmental stimuli, e.g., temperature, nutrients and oxygen availability, and the presence of predators or parasites. The exposure to stresses during development can be deleterious or beneficial to the adults. For example, changes during development influence adulthood disease propensity in humans [2]. The adult *Caenorhabditis elegans* that transiently passed through the stress-induced dauer larval stage exhibit an extended lifespan compared to animals with normal development [3]. Environmental conditions, i.e., temperature, and oxygen and carbon dioxide concentrations [4,5], were shown to direct the development of nematode *Strongyloides ratti* to distinct parasitic or free-living adults whose aging rates can differ by 30-fold [6]. Biotic factors can also induce dramatic developmental alterations. For example, in response to waterborne cues from predators, some *Daphnia* species grow protective helmets [7]. Microorganisms, which are a major environmental component, also have an important impact on development. The effect of microorganisms on development was established by comparing axenic animals against animals colonized with microbes. In fruit fly and zebrafish, axenic development can result in slower development, reduced fecundity, and/or early death [8,9]. Axenic *C. elegans* have a slower development, reduced fecundity, and increased lifespan [10]. In mammals, microorganisms can affect the maturation of the mucosal immune system [11], modulate the proliferation and the differentiation of intestinal epithelia [12], regulate angiogenesis [13], and play a key role in the extraction and the processing of nutrients [14].

Microorganisms are classified as commensals when they are innocuous and/or beneficial for the host, and pathogens when they are harmful to the host [15]. It should be noted that pathogens most frequently do not kill their hosts, but decrease host fitness and increase host susceptibility to environmental challenges. Adult hosts mount different responses to various types of microorganisms, which have a profound effect on the hosts themselves. While it is clear that presence or absence of microorganisms can have an important impact on development, how exposure to different types of microorganism, i.e. innocuous and pathogenic, during development affects adult animals health, stress resistance and longevity is unknown. Thus, in this study we examined the consequences of exposure of *C. elegans* nematodes during development to different bacterial strains. *C. elegans* was chosen because it is a well-studied animal model for aging, development, and host-pathogen interactions, and isogenic populations can be generated and maintained. The bacterium *Escherichia coli* was chosen because it is a well-studied model organism and a species with a wide virulence spectrum, ranging from innocuous to highly pathogenic in diverse hosts. It is also of particular importance that the standard laboratory food for *C. elegans* is one *E. coli* strain, OP50 [16,17]. Survival/longevity of nematodes fed with OP50 is considered as the baseline for all *C. elegans* studies. *E. coli* strains are among the first colonizers of the intestinal tract of newborn warm-blooded animals. *E. coli* strains are mainly harmless commensals, but some strains are able to cause intestinal and extra-intestinal diseases, which by their frequency and potential severity cause considerable morbidity and significant health-care costs [18]. We previously demonstrated that *E. coli* pathogenic strains can significantly reduce the lifespan of the adult *C. elegans*[19,20], and that *E. coli* pathogenicity factors are responsible for the observed lifespan reduction.

For this study, *C. elegans* nematodes were fed on different bacterial strains during development and then with the same or different bacteria as adults. We found that nematodes developed on a pathogen lived longer when exposed to pathogens as adults than nematodes that were developed on an innocuous strain. Some of the findings observed with the pathogenic *E. coli* were confirmed using a second pathogen, a Gram-positive *Enterococcus faecalis*. The observed increase in nematode lifespans is controlled by the DBL-1 transforming growth factor beta-like, DAF-2/DAF-16 insulin-like signaling, and partially by the PMK-1 p38 MAP kinase pathways. Developmental exposure to one pathogen increases resistance of adult nematodes to the same and other pathogens, as well as to heat shock. Hence, development on pathogens has a hormetic effect on adult nematodes. Hormesis is a response of organisms to low doses of a damaging environmental factor resulting in resistance to higher doses of the same, but also to other stressful environmental factors [21,22]. The possible consequences of the observed hormetic effect in natural environments are also discussed.

## Results

For this study, we used the reference OP50 and pathogenic 536 *E. coli* strains, as well as *Enterococcus faecalis* OG1RF strain. The innocuity of the OP50 strain was attested by observation that the mean lifespan of the *C. elegans fer-15* nematodes was the same on the live and UV-killed OP50 bacteria [20]. *fer-15* is a derivative of the *C. elegans* N2 strain, which we used in the present study. Strain 536 is an uropathogenic *E. coli* isolated from a patient with acute pyelonephritis [23]. Pathogenicity of the 536 strain was previously demonstrated in the mice sepsis model and in *C. elegans*[19]. Adult *fer-15* nematodes exposed to strain 536 have a 50% shorter mean lifespan compared to nematodes exposed to the OP50 strain [19]. Deletion of pathogenicity islands II and III significantly reduces the pathogenicity of strain 536 in *C. elegans fer-15*[19], while it does not reduce the bacterial growth rates nor resistance to biologically relevant stressors [24]. This shows that strain 536 reduces *C. elegans* lifespan because of its pathogenicity factors and not because it does not provide an adequate food source.

N2 nematodes were developed on one bacterial strain and late L4 stage nematodes were subsequently maintained on the same strain or transferred to a different one. For this reason, the designation of each experimental condition contains: (i) the name of the strain on which larvae were developed and (ii) the name of the strain to which adult nematodes were exposed. For example, OP50/536 indicates that nematodes were developed on OP50 and then transferred onto strain 536. All experiments were performed at 25°C.

### Lifespan of nematode populations developed on different bacterial strains

First, we compared the developmental success, defined as the fraction of nematodes reaching adulthood from an initial number of eggs, for *C. elegans* fed either on the OP50 or on the pathogenic 536 strain. We observed no significant difference (unpaired t-test, p > 0.7549) in the fraction of nematodes that reached adulthood between these two experimental conditions (mean ± s.e.m.: 91.6% ± 1.7% developmental success on OP50, n = 263; 92.3% ± 1.6% on 536, n = 262). Therefore, it can be concluded that nematode development is robust and that larvae survival is not affected by the pathogen.

**Figure 1:**
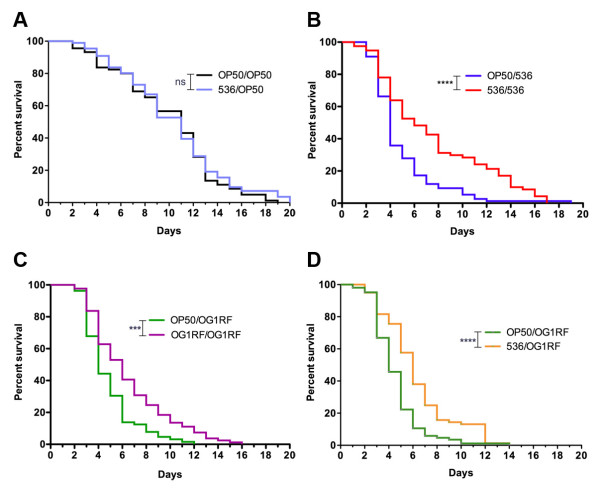
**Survival of *C. elegans *developed on different *E. coli *strains.** (**A**) Survival of N2 nematodes to the innocuous *E. coli* OP50 strain following development on the pathogenic 536 strain (536/OP50 n = 93 and OP50/OP50 n = 90, independent replicates N = 3). There is no difference in nematodes survival despite the difference in developmental condition indicating that development on the pathogenic 536 strain has no deleterious effect on survival. (**B**) Survival of N2 nematodes to *E. coli* 536 strain following development on the pathogenic 536 strain (536/536 n = 80 and OP50/536 n = 80, N = 7). N2 nematodes had a significantly greater survival on strain 536 following development on 536 than on OP50 strains. (**C**) Survival of N2 nematodes to *E. faecalis* OG1RF strain following development on *E. faecalis* OG1RF (OG1RF/OG1RF n = 86, OP50/OG1RF n = 81, N = 3). As seen with *E. coli* 536, development on *E. faecalis* OG1RF prior to adults exposure to the same strain significantly increases nematodes survival compared to those developed on OP50 strain. (**D**) Survival of N2 nematodes to *E. faecalis* OG1RF strain following development on *E. coli* 536 (536/OG1RF n = 100 and OP50/OG1RF n = 104, N = 3). Development on *E. coli* 536 significantly increases survival to *E. faecalis* OG1RF strain in a similar manner as development on *E. faecalis* OG1RF, and is thus not due to adaptation to one bacterial strain during nematode development. Graphed: ns: not significant, *: p < 0.05, **: p < 0.01, ***: p < 0.001, ****: p < 0.0001.

Second, we investigated the lifespan of adult nematodes on the innocuous OP50 strain after they were developed on OP50 or on the pathogenic 536 strain. For these experiments, we eliminated bacteria from the L4 nematodes by antibiotic treatment and then transferred them on OP50 strain. No difference (Gehan-Breslow-Wilcoxon Test (GBWT), p = 0.7334) in lifespan was observed between OP50/OP50 and 536/OP50 nematodes (Figure 1A). These results suggest that development on pathogens produce healthy adult nematodes that, when exposed to non-stressful conditions, have identical lifespan to that of nematodes exposed to the innocuous bacterial strain throughout life.

Third, we investigated the lifespan of adult nematodes on the pathogenic 536 strain after they were developed on OP50 or on the 536 strain. As shown in Figure 1B, the exposure of nematodes to the pathogen during development increased median lifespan (TD_50_) by 50% (OP50/536 TD_50_ = 4 days and 536/536 TD_50_ = 6 days, GBWT, p < 0.0001). The increase of survival was also significant during the reproductive period (days 1 to 4) (OP50/536 survival at day 4 S_D4_ = 35.8% ± 5.5% to 536/536 S_D4_ = 67.9% ± 5.3%, chi-square test, p = 1.18×10^-7^). The beneficial effect of developmental exposure to pathogen on the adult nematodes’ lifespan was also observed with Gram-positive *Enterococcus faecalis* OG1RF strain, i.e., OG1RF/OG1RF nematodes had significantly increased (GBWT, p = 0.0007) median lifespan compared to OP50/OG1RF nematodes (Figure 1C).

Observed lifespan extension for nematodes developed and maintained through adulthood on pathogens could be explained by adaptation to a particular type of stress encountered during development, which renders adult nematodes more resistant to the same stress, i.e., exposure to the pathogenic 536 strain. In order to verify this possibility, we developed nematodes on *E. coli* 536 strain, eliminated the bacteria from the L4 nematodes by antibiotic treatment and transferred them on the *E. faecalis* OG1RF strain*.* Development on *E. coli* 536 strain significantly (GBWT, p < 0.0001) increased lifespan of adults exposed to the *E. faecalis* OG1RF strain (Figure 1D) to the same extent as development on OG1RF itself did (Figure 1C). These results show that the beneficial effect of developmental exposure to pathogens on nematode lifespan was not due to adaptation to one particular type of strain or stress, but also provides protection against other bacterial strains. Developmental exposure to Gram-positive and -negative bacterial strains has a cross protective effect, indicating that the recognition of membrane lipopolysaccharides, which are present at the surface of Gram-negative but not of the Gram-positive bacteria, is not involved in this phenomenon.

### *C. elegans* signaling pathways involved in lifespan extension following development on pathogens

**Figure 2:**
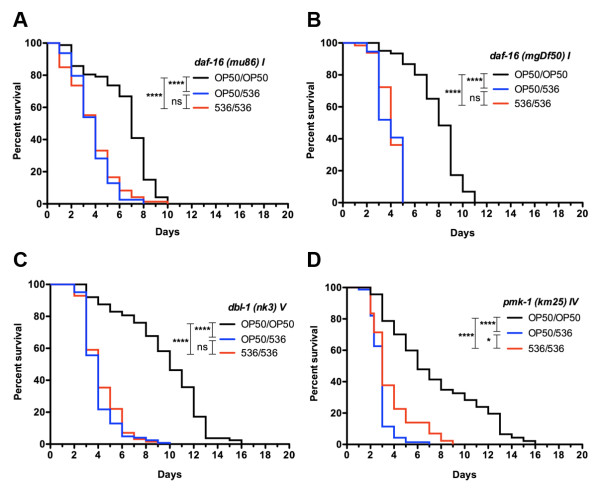
*** C. elegans *signaling pathways involved in developmental modulation of longevity. ** Survival of *C. elegans* mutants to *E. coli* 536 pathogen following development on strains 536 (536/536) or OP50 (OP50/536) and life through OP50 control (OP50/OP50). (**A** &**B**) *C. elegans daf-16* mutants allele mu86 (536/536 n = 80, OP50/536 n = 80, and OP50/OP50 n = 80, independent repeats N = 4) and mgDf50 (536/536 n = 65, OP50/536 n = 56, and OP50/OP50 n = 61, N = 3), (**C**) *C. elegans dbl-1* mutant (536/536 n = 127, OP50/536 n = 124, and OP50/OP50 n = 88, N = 4), and (**D**) *C. elegans pmk-1* mutant (536/536 n = 80, OP50/536 n = 78, and OP50/OP50 n = 77, N = 8). No differential survival of *daf-16* and *dbl-1* nematodes to *E. coli* 536 based on developmental conditions was observed, while a small but statistically significant difference in survival to *E. coli* 536 strain was observed for *C. elegans pmk-1* mutant depending on developmental conditions. Graphed: ns: not significant, *: p < 0.05, **: p < 0.01, ***: p < 0.001, ****: p < 0.0001.

We examined the role of different immune signaling pathways in the nematode lifespan extension following development on pathogens. We first evaluated if two signaling pathways implicated in *C. elegans* development and in adult immunity are involved in the observed development-induced modulation of the adult lifespan: the DAF-2/DAF-16 insulin-like pathway [25] and the DBL-1 transforming growth factor ß-like (TGFß-like) pathway [26]. These two signaling pathways function from the first larval stages of nematode development, are involved in the signaling and regulation of the immune response to pathogens and in stress responses to environmental factors in adults [27]. When DAF-2/DAF-16 insulin-like or TGFß-like pathways were inactivated, there were no significant lifespan differences between adult populations exposed to the pathogen after development on OP50 or on 536 (Figure 2A-C, GBWT, *daf-16* mutants p = 0.9368 and p = 0.4082 for alleles mu86 and mgDf50, respectively, *dbl-1* mutant p = 0.1985). We also tested the implication of another innate immunity pathway, the p38 MAP kinase pathway via the *pmk-1* mutant [28]. The difference in lifespan of the *pmk-1* nematodes between OP50/536 and 536/536 conditions was reduced, but still significantly different (GBWT, p = 0.0106, Figure 2D). The mean lifespan of *pmk-1* nematodes was 25% shorter compared to the two other innate immunity pathways mutants, which indicates that the lack of differential survival of *daf-16* and *dbl-1* mutants was not due to the saturation of our assay with maximal killing. Thus, it can be concluded that the observed reduction of the mortality of adult N2 nematodes (Figure 1B) is mainly modulated by the DAF-2/DAF-16 insulin-like pathway and TGFß-like pathway. Therefore, the observed modulation of adult nematodes lifespan by developmental exposure to pathogens is likely a genetically controlled nematode response. 

### *C. elegans* resistance to heat shock following development on pathogens

Induction of the heat shock response in *C. elegans* limits the accumulation of damaged proteins, increases stress resistance, resistance to pathogens, and lifespan [29-31]. It was also reported that the capacity to induce the heat shock response early in life correlates positively with the nematode life expectancy [32]. For these reasons, we tested whether development on pathogens (i) increases the capacity of adult nematodes to resist heat shock, and (ii) if it results in the induction of the heat shock response.

**Figure 3:**
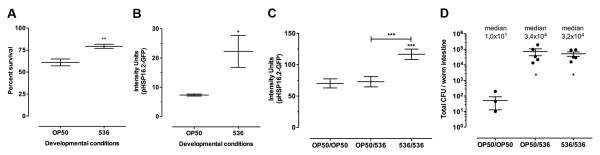
**Heat-shock resistance and *hsp-16.2 *induction in *C. elegans *developed on different *E. coli *strains.** (**A**) Survival proportion of nematodes developed on OP50 or 536 *E. coli* strains after a 10 h heat shock at 35°C. Following development on strain 536, 20% more nematodes survived the 10 h heat shock treatment compared to nematodes developed on strain OP50. (**B**) Level of fluorescence of the *hsp-16.2*::GFP reporter in young adult nematodes (day 0) developed on *E. coli* 536 pathogen (n=75) or OP50 strain (n=75). (**C**) Two day-old adult nematodes exposed to the 536 pathogenic strain had a higher level of induction of *hsp-16.2* following development on strain 536 (536/536 n=150) than those developed on strain OP50 (OP50/536 n=88) and those maintained life through on strain OP50 (OP50/OP50 n=98). (**D**) Quantity of live bacterial cells in the intestinal tract of 2-day old nematodes exposed to *E. coli* 536 following development on strains 536 (536/536 n=5) or OP50 (OP50/536 n=5) and controls maintained life through on strain OP50 (OP50/OP50 n=5). The observed difference in the level of *hsp-16.2* induction cannot be correlated with the amount of live bacteria colonizing the nematode intestinal tracts as there was no significant difference in the mean bacterial density in the intestine of 2-day old nematodes exposed to *E. coli* 536 independently of whether they were developed on strains OP50 or 536. Graphed: mean ± s.e.m., *: p<0.05, **: p<0.01, ***: p<0.001.

First, we found that nematodes developed on pathogens resist significantly (unpaired t-test, p = 0.0009) better than nematodes developed on OP50 to a 10 h heat shock at 35°C (Figure 3A). Second, we measured the induction of the heat shock response in these two groups of nematodes. For this, we used a *C. elegans* strain carrying a fusion of a gene coding for the Green Fluorescent Protein (GFP) with the *hsp-16.2* gene promoter. The expression of *hsp-16.2* gene, which codes for a chaperon, is regulated by the Heat Shock Factor 1 (HSF-1) and DAF-16. We observed that development on the 536 strain, compared to development on OP50, induced a significant (unpaired t-test, p = 0.0138) upregulation of *hsp-16.2* expression in L4 nematodes (Figure 3B). The observed difference was also highly significant (Tukey’s Multiple Comparison Test (TMCT), p < 0.001) in 2-day old nematodes (Figure 3C). The difference in *hsp-16.2* induction cannot be explained by the amount of live bacteria in the intestinal tract, as there was no significant difference in the number of live bacteria in the intestine of 2-day old nematodes exposed to the 536 strain whether they were developed on OP50 or 536 (Figure 3D). Therefore, the induction of *hsp-16.2* depends on developmental and adulthood conditions, and correlates with the resistance to heath shock and the lifespan extension of adult nematodes exposed to pathogens, relative to the nematodes developed on the OP50 strain.

## Discussion

We showed that the development of nematodes on bacterial pathogens increases the lifespan of adult nematodes exposed to the same or different pathogens, and increases resistance of nematodes to heat shock. It is important to note that such increased robustness of nematodes developed on pathogens is not due to the culling of weak individuals from the population, as the same fraction of nematode larvae reached adulthood following development on pathogenic and innocuous strains. In addition, there was no difference in the lifespan of adult nematodes on the innocuous OP50 strain after they developed on OP50 or on the pathogenic 536 strain, which indicates that development on a pathogen does not damage larvae.

Hosts can defend themselves from pathogens using a variety of strategies [33]. The avoidance strategy minimizes the risk of infection with pathogen and economizes the nematode’s resources, which otherwise would be used for the activation of its defenses [34]. The avoidance strategy requires that host first detects pathogens. In nature, *C. elegans* is predominantly found in decaying organic matter where it feeds on microorganisms, which can be good or harmful food [35]. *C. elegans* possesses a complex chemosensory neuronal system that allows discrimination between these two groups of microorganisms based on the detection of a number of volatile odorant and water-soluble compounds [36-38]. For example, nematodes are repulsed by a cyclic lipodepsipentapeptide produced by *Serratia marcescens,* a bacterium that is harmful to nematodes [39]. *C. elegans* also has the capacity to identify pathogens and harmful xenobiotics by monitoring its essential cellular activities, such as protein translation or oxidative respiration. Perturbations of these functions induce aversion behavior and defense mechanisms [37]. We did not study the molecular basis of the discrimination between different bacterial strains by nematodes. However, the fact that the observed hormetic effect is not bacterial species specific suggests that it is more likely induced by the nematode surveillance of its own essential cellular functions, rather than by recognition of the specific compounds produced by bacteria.

Nematodes also have a capacity to memorize, into adulthood, environmental cues encountered during early life [40]. Can avoidance behavior based on memory of past conditions contribute to the longer lifespan of nematodes developed on a pathogen and exposed to the same pathogen as adults, compared to nematodes developed on an innocuous strain and exposed to pathogens as adults? This is probably not the case in our study because independently of the developmental conditions, nematodes have the same amount of the 536 pathogenic bacteria in their intestine, which indicates that 536/536 nematodes do not live longer than OP50/536 nematodes because they avoid pathogens and eat less. The fact that the intestinal pathogen burden is independent of developmental conditions also suggests that, in our case, the longer lifespan of nematodes developed on, and then exposed to, pathogens as adults were not the result of enhanced capacity to kill bacteria, but likely due to the increased tolerance to the damages inflicted by pathogenic bacteria. Tolerance is defined as the ability to limit negative impact on hosts by increasing cellular repair and maintenance capacity, without affecting pathogen burden [33,41]. Increased investment in repair and maintenance function often comes with a cost for the host. In our study there is no evidence of such cost as, nematodes developed on pathogen and then transferred to non-stressful conditions, i.e., innocuous bacterial strain, have lifespans identical to the lifespan of nematodes exposed to the innocuous bacterial strain throughout life. However, it cannot be excluded that the cost of tolerance becomes evident under experimental conditions that are closer to those found in natural environments.

From a molecular perspective, increased tolerance to pathogens of the nematodes developed on pathogens could be, at least partially, due to the induction of the heat shock response. We observed that adult nematodes developed on pathogens induce higher levels of expression of the *hsp-16.2* gene than nematodes developed on an innocuous strain. The *hsp-16.2* gene expression was shown to be regulated by DAF-16 and HSF-1 transcription factors in the N2 nematodes [42-44]. It was previously observed that the expression level of *hsp-16.2* in individual nematodes exposed to a mild heat shock was predictive both of thermotolerance and lifespan [32,45]. Overproduction of HSP-16.2 also suppresses beta-amyloid peptide toxicity [46]. There are many other examples of the correlation between increased stress resistance and longevity in nematodes [47]. For example, the DAF-2/DAF-16 insulin-like pathway mediates stress resistance, longevity and pathogen resistance in *C. elegans*[48]. This supports our hypothesis that the nematode’s response to developmental conditions observed in this study is genetically controlled by the DAF-2/DAF-16 insulin-like pathway. However, it was reported that *daf-16* and *dbl-1* mutants have an increased amount of live bacteria in their intestine compared to N2 nematodes when maintained on *E. coli* OP50 [49]. On the contrary, a *pmk-1* mutant has similar amount of live bacteria in its intestine when compared to that of N2 nematodes. Thus, the absence of increased survival for *daf-16* and *dbl-1* mutants could also be due to an increased intestinal bacterial load following development on the pathogen, counterbalancing the beneficial effect of early pathogen exposure. Further investigation of these signaling pathways will be necessary to determine the direct or indirect involvement of these pathways in the control of nematode response to developmental exposure to bacterial pathogens.

Observed effect of developmental conditions on the adult lifespan and stress resistance could be described as hormetic. Hormetic response in *C. elegans*, i.e., increased stress resistance and/or increased lifespan, was shown to be induced with sub-lethal abiotic stresses, like oxidative and thermal stress [47,50]. However, all forms of stress, e.g. UV or ionizing radiation, do not produce hormetic effects in *C. elegans*[51]. Our study shows that some biotic factors, i.e., bacterial pathogens, can be added to the list of known hormetic stressors. One possible explanation for the hormetic effects of exposure to low levels of stress is that the level of response to stress is more than necessary to repair the damage caused, thus increasing the nematode defense capacity and allowing tolerance to future stresses. A particularity of our study, contrary to other above-mentioned studies of the hormetic effect of different stressors on *C. elegans*, is that we induced hormetic effect by exposing nematodes to bacterial pathogens during development as stressors. We hypothesize that hormetic effects of early exposure to bacteria are common in nature as they were observed in a variety of organisms. For example, priming of innate immunity by pathogens has been reported for invertebrates such as *C. elegans*[52,53] and insects [54]. In some cases, immune priming can even be trans-generational, as observed for the mealworm beetle *Tenebrio molitor*[55]*.* It was shown that the presence of bacteria during the first week of the *Drosophila melanogaster* adult life increases the flies lifespan [56]. The early life interactions between human host and bacteria, which largely occur through the colonization of the newborn intestine, are also increasingly recognized as being critical for the maturation of human immune system as well as for the metabolic homeostasis of the host [57]. Newborn babies whose intestinal colonization was modified by the mode of delivery (natural vs. Cesarean section) or by antibiotic treatments, show a delay in immune response maturation [58]. Early exposure of children, up to five years of age, to non-septic bacteria-rich environments, has a significant protective effect against the onset of allergies [59]. Epidemiologic studies also show an increased occurrence of autoimmune diseases in human populations in “clean” environments [60]. These observations constitute the basis of the “hygiene hypothesis,” according to which the lack of exposure to microbes due to high hygienic conditions commonly found in the Western world prevents correct maturation of the immune system and predisposes individuals to allergies and other immune diseases. These observations also correspond to the programming concept, which refers to stimuli that during critical periods of development may “program” the long-term structure or function of an organism [61]. Our data establish that, at least in the *C. elegans* model, intentional administration of particular bacterial strains during early life could indeed modulate disease susceptibility during adulthood.

Our study strongly suggests that variations induced during development are maintained in genetically identical adult nematodes, as it was previously reported for the nematodes that transiently passed through the stress-induced dauer larval stage [3]. In *C. elegans*, the molecular basis of such life-long memory of modifications of developmental transcription profiles is mediated by histone modifications [3]. Whether histone modifications are also responsible for the phenotypes we observed remains to be determined.

Although natural selection might favor improved sensing and response mechanisms, adaptation could also result in the emergence of more sophisticated response strategies. Developmental plasticity allows for the production of phenotypically diverse offspring in populations of genetically identical individuals, which increases the probability of survival in changing and challenging environments. The evolution of the ability to mount such predictive developmental modifications depends on a number of features, such as the cost of developmental plasticity, ability to correctly detect and interpret environmental cues that depends on the frequency by which various environmental conditions are encountered, and finally on the long term fitness advantage provided. Therefore, as described for *E. coli* plus *Saccharomyces cerevisiae*[62], and *C. elegans*[52], the observed phenomenon could extend beyond merely sensing and responding immediately to a given stimulus and could contribute to a predictive response strategy that uses the appearance of a stimulus as a cue that future conditions might be stressful.

## Conclusions

Our study shows that development on pathogens has a hormetic effect on adult nematodes, as it results in increased resistance to different pathogens and to heat shock. Such developmental plasticity of *C. elegans* nematodes, which are self-fertilizing homozygous animals producing offspring with negligible genetic variation, could increase the probability of survival in changing environments.

## Methods

### Nematode and bacterial strains

*C. elegans* N2 (ancestral) strain used for all experiments unless otherwise indicated was kindly provided by J.J. Ewbank (Marseille, France). Strain TJ375 (gpIs1[*hsp-16.2*::GFP]) and mutant strains CF1038 (*daf-16* (mu86) I), GR1307 (*daf-16* (mgDf50) I), NU3 (*dbl-1* (nk3) V), and KU25 (*pmk-1*(km25) IV) were obtained from the Caenorhabditis Genetics Center (Minneapolis, Minnesota, USA). N2 control was included in every experiment with nematode mutants.

*E. coli* strains used in this study were pathogenic strain 536 [23], and the uracil deficient strain OP50 [16]. Genotypic and phenotypic characterization of these strains was described previously [19]. *Enterococcus faecalis* strain OG1RF [63] (previously named *Streptococcus faecalis* OG1-RF1) was kindly provided by Dr. P. Courvalin (Paris, France).

### Nematode maintenance and synchronization

Nematodes were maintained at 25°C on nematode growth medium (NGM) agar plates seeded with 0.1 mL LB grown stationary phase bacterial culture, and incubated 18 h at 37°C to densities of 7.3 × 10^9^ ± 3 × 10^8^, 1.4 × 10^10^ ± 5 × 10^9^, and 1.5 × 10^10^ ± 2 × 10^9^ CFU/plate for *E. coli* OP50, 536, and *E. faecalis* OG1RF, respectively. Age-synchronized populations of nematodes were initiated from eggs recovered following sodium hydroxide (0.5 M final) and sodium hypochlorite (0.96% final) treatment of gravid adults maintained at 25°C and fed *E. coli* OP50. All assays were carried out at 25°C with nematodes synchronized a second time at the end of development by selecting exclusively nematodes at the end of the 4^th^ larval (L4) stage based on vulva morphology.

### Survival assays

Survival assays were carried out at least in triplicate. For survival assays, all nematode strains were developed and maintained at 25°C, transferred onto new plates every day during the first 5 days to eliminate progeny, and every 2–3 days thereafter. Dead nematodes were scored every 24 h. A nematode was considered dead when it failed to move spontaneously or respond to a gentle touch with a platinum wire. Nematodes buried in the agar or on the sides of the plates were censured from the analyses. Lifespan was measured as the time from the end of L4 larval stage (beginning of adulthood) until death.

### Elimination of bacteria from nematodes intestine

When experiments involved nematodes developed on *E. coli* 536 or *E. faecalis* OG1RF and then transfer to a different bacteria strain, L4 stage nematodes were washed and treated with antibiotics to remove the initial strain from their intestinal tract. Nematodes were washed in M9 minimal salts buffer to remove excess bacteria from their surface and then incubated in NGM for 1 h to allow them to expurgate intestinal bacteria. Nematodes were then treated for 1 h with 20 μg/ml Polymyxin B antibiotic in M9 minimal salts buffer. Finally, nematodes were washed in M9 minimal salts buffer and transferred on plates containing the appropriate bacterial strain. The effect of antibiotic treatment on nematode survival was taken into account by applying the treatment to all nematode populations involved in the assays. For survival experiments, treated nematodes were regularly checked for the absence of the initial bacterial strain. A few nematodes were sampled from the survival assay, crushed in a Dounce homogenizer and the extract plated on LB agar media for colony observation (see below quantification of live bacterial cells in nematode intestine for detail on this procedure). Colony morphology and color allowed for direct visual discrimination between the different bacterial strains used in this study.

### Measurements of nematode heat shock resistance

L4 nematodes developed on OP50 or 536 were washed in Polymyxin B antibiotic (see above elimination of bacteria from nematodes intestine). Then, nematodes were transferred onto OP50 lawn in order to measure heat shock resistance using nematodes that have the same bacterial strain in their intestines. After allowing nematodes to recover for 12 h at 25°C, they were transferred for 10 h at 35°C. Dead nematodes were scored at the end of the 10 h incubation at 35°C. This assay was performed in triplicate with internal triplicate controls.

### Measurements of nematode heat shock protein (HSP) expression

HSP expression measurement assays were carried out with *C. elegans* strain TJ375 carrying a fluorescent reporter under the control of the *hsp-16.2* promoter (gpIs1*hsp-16-2*::GFP]). Nematodes were collected with cold (4°C) sterile water, fixed immediately by addition of an equal volume of cold 2% formaldehyde in 2 × Phosphate Buffered Saline (PBS), and incubated 10 min on ice. Fixed nematodes were collected by gravity sedimentation and washed in cold 1× PBS before being loaded into a 96-well plate kept at 4°C in the dark until analyzed. Two-day old adults were separated from their progeny on the first and second day by gravity sedimentation in 15 ml falcon tubes for 2 min in M9 minimal salts buffer, the larvae being removed with the supernatant. The absence of eggs and larvae was verified under a dissecting microscope. Gene reporter levels were quantified with the COPAS Biosort (Union Biometrica) as described in [64]. Briefly, worms were analyzed for size (TOF) and green (GFP) fluorescence. Raw data were filtered on the TOF (200–1000) to exclude dust, bubbles, and aggregated worms. Fluorescence data was acquired from two independent experiments, including internal replicates.

### Quantification of live bacterial cells in nematode intestine

Two-day old nematodes were handled at 4°C to stop defecation. Nematodes were transferred to a new plate without bacteria, recovered in 1.5 ml 10^-2^ M MgSO_4_, and vortexed 30 seconds to remove excess bacteria from nematode cuticles. Then, individual nematodes were again transferred to a new plate without bacteria and rubbed across the plate to remove all bacteria from the cuticle. Nematodes were then individually crushed in a Dounce homogeneizer with the fitted mortar (B) in 10^-2^ M MgSO_4_. The amount of live bacteria was determined by plating of appropriate dilutions on LB agar. After overnight incubation at 37°C, grown colonies were counted.

### Statistical analyses and figures

Statistical analyses and graphic displays were made using Prism 5.0d from GraphPad Software, Inc. Measurement assays were analyzed by unpaired t-test for comparison of two groups or, when more than two groups were involved, using 1-way analysis of variance (ANOVA) followed by Tukey’s Multiple Comparison Test (TMCT) also known as Tukey-Kramer test, comparing all pairs of group and allowing for unequal sample sizes. Survival assays were analyzed using the Gehan-Breslow-Wilcoxon Test (GBWT) comparing conditions by pairs and allowing for unequal hazard ratios. Data presented are mean ± s.e.m., unless otherwise indicated. For survival assays, a typical, representative experiment is presented, the absence of significant difference between replicates was verified using GBWT .

## Competing interests

The authors declare that they have no competing interests.

## Authors’ contributions

ML, TM, XM and IM designed experiments, analyzed and interpreted data, and drafted the manuscript. ML, TM, XM and DFA performed experiments. All authors read and approved the final manuscript.
